# Biocompatibility of CAD/CAM Milled Dental Restorative Materials: A Systematic Review from In Vitro Studies

**DOI:** 10.3390/ma18184323

**Published:** 2025-09-16

**Authors:** Andrzej Małysa, Janka Jenčová, Joanna Weżgowiec

**Affiliations:** 1 Department of Experimental Dentistry, Wroclaw Medical University, 50-425 Wroclaw, Poland; 2 Clinic of Stomatology and Maxillofacial Surgery, Faculty of Medicine, University Pavol Josef Safarik and Akademia Kosice, 041 90 Kosice, Slovakia; janka.jencova@upjs.sk

**Keywords:** CAD/CAM, milling, dental restoration, fixed dental prosthesis, dental materials, cytotoxicity

## Abstract

The introduction of new materials for fixed dental prostheses (FDPs) necessitates the demonstration of excellent physical and mechanical properties alongside biocompatibility to fulfill their intended function. This systematic literature review sought to assess studies that compare the biocompatibility and toxicity of CAD/CAM milling dental materials. PubMed, Scopus, and Web of Science were screened to locate English-language, full-text articles published between 1 January 2014, and 31 October 2024. Initially, 1050 records were identified, and after a thorough screening, 78 full texts were evaluated, resulting in the inclusion of 33 studies. The reports were heterogeneous regarding materials, cell lines, and methodologies; thus, comparisons were made within studies rather than between them. The majority of the reviewed studies indicated that CAD/CAM milled materials generally exhibited lower toxicity than conventionally fabricated materials. Additionally, several novel experimental CAD/CAM materials demonstrated promising biocompatibility results.

## 1. Introduction

The computer-aided design and computer-aided manufacturing (CAD/CAM) system is a series of technologies that allows for digital design and production of all works with incredible accuracy and precision. Currently, there are two known divisions of this technology, milling (subtractive) and printing (additive), both widely used in dentistry [1]. Each of these two methods has its advantages and disadvantages. The presented systematic review focuses on subtractive technology, which is characterized by work on fully hardened materials such as polymers, giving an incredible advantage of working on technically processed material [2], hypothetically and, by definition, free from monomers exhibiting significant cytotoxicity. Milling technology also processes materials such as lithium disilicate or zirconium that require crystallization as the next stage. However, ceramic materials are assumed to be more biocompatibly advanced compared to polymers [3,4,5,6].

The proper selection of the material is particularly important for the success of dental reconstructions. Depending on the degree of damage to the hard tissues of the tooth or purely aesthetic indications, reconstructions limited to the individual tooth are used, such as crowns, veneers, inlays, or onlays [3,4,5]. In the case of extensive tooth loss, bridges can be used as a fixed dental prosthesis, depending on the selected treatment procedure. The current direction of digital dentistry development is largely based on new, innovative materials. In prosthetics, the following are mainly used: zirconium, metal, polymethyl methacrylate (PMMA), glass ceramics, and polyetheretherketone (PEEK). Commonly, CAD/CAM polymer materials are divided into three basic groups: first, resin-filled hybrid ceramics based on “polymer-infiltrated ceramic network” (PICN); second, polymers based on PMMA resins with low inorganic filler contents; and last, polymers based on different N-(3,4-dihydroxyphenethyl)methacrylamide (DMA), such as bisphenol A diglycidyl dimethacrylate (BisGMA), urethane dimethacrylate (UDMA), and bisphenol A dimethacrylate (BisDMA), and which feature a high content (61–86 wt%) of inorganic fillers [4]. It is also worth mentioning the continuous design of new materials with properties primarily focused on high biocompatibility, containing hydroxyapatite or bioactive glass [6,7,8,9]. Currently designed materials used in restorative dentistry not only restore damaged hard tissue of teeth, but also have the potential to reduce plaque formation or secondary caries development [7,8,10].

It is worth mentioning that biocompatibility is a broader assessment that includes evaluating cytotoxicity, along with other factors like immunogenicity and hemocompatibility, to determine the overall safety and efficacy of a material for dental use [8]. Cytotoxicity refers to the ability of certain substances to cause damage to or kill living cells [9]. It is assessed in the context of testing the safety of currently used materials or developing new ones. Cytotoxic effects cause disruption of cellular functions, such as proliferation, and result in cell death by different mechanisms, e.g., necrosis (uncontrolled cell death) and apoptosis (programmed cell death) [8,10]. This review focuses on any effects that have an impact on a broad term of biocompatibility.

It should be emphasized that nowadays most branches of modern dentistry are based on CAD/CAM materials regardless of whether they are ceramics or polymers [11,12,13]. Regardless of their characteristic structure, they tend to possibly release various substances into the oral cavity, raising doubts about the safety of their use [9,11,12,13,14]. Additionally, over time, all dental structures degrade in a very harsh oral cavity environment. Mechanical damage, enzymatic hydrolysis, and increased water sorption through increased porosity will lead to the release of different, possibly harmful components [5,7,15,16]. Currently, interesting alternatives to the conventional method of manufacturing are offered by CAD/CAM technologies in combination with industrially manufactured polymer discs [17]. While these materials are processed indirectly, the digital workflow offers various advantages such as a fast manufacturing process and the opportunity to duplicate the restoration in case of failure [10,16].

This systematic review was conducted following the PRISMA 2020 (Preferred Reporting Items for Systematic Reviews and Meta-Analyses) guidelines used to collect and report data [18]. This systematic review aimed to select studies that examined the toxic effects of various dental prosthetic milled materials used in the fixed reconstruction. This review aims to determine and compare the results of various in vitro experimental laboratory research focused on the cytotoxicity of commercially available and experimental materials.

## 2. Materials and Methods

The primary outcome for assessing the biocompatibility of CAD/CAM milled dental materials focuses on their influence on cell viability by measuring the ability to survive and function in the presence of the material. The selected studies evaluated cytotoxic effects the material may have on cells, including any inflammatory or immune responses in cells when exposed to the material. The research was conducted by searching the Scopus, Web of Science, and PubMed databases to identify the relevant full-text articles published in English between 1 January 2014 and 31 October 2024. Searching the literature was performed by combining each of the following keywords: (1) milled OR (2) CAD/CAM; with each of the following keywords: (I) denture OR (II) restoration OR (III) prosthesis AND each of the following keywords: (A) cytotoxicity, (B) biocompatibility. Bibliographic references of accepted studies were hand-searched to retrieve articles aimed at the identification of potentially relevant papers [17]. The authors manually screened each record. Tools to automate the collection process were not used. Inclusion and exclusion criteria were defined according to the PICOS (Population, Intervention, Comparison, Outcomes, and Study Design) approach and are listed in Table 1. Two authors (J.W. and A.M.) were independently involved in the literature identification and record-screening procedure. After removing the duplicates from the records identified in different databases, the titles and abstracts of the remaining records were screened based on the inclusion and exclusion criteria. For a final evaluation of eligibility, two authors performed an independent assessment of the full text of the selected articles, which was critically reviewed (J.W.). Any discrepancies between the reviewers at this stage were discussed in detail. If consensus could not be reached, a third reviewer (J.J.) was consulted to provide an independent assessment and facilitate a final decision. This process ensured that all relevant studies were considered, and the selection was as inclusive and unbiased as possible. The use of a third reviewer as an arbitrator not only resolved conflicts but also enhanced the overall reliability of our study selection process, contributing to the robustness of the systematic review.

The information collected from the qualified articles included sample size and type of tested milled materials used in CAD/CAM, cell line types, type of biocompatibility assay, exposure time, and side effect detection. Attention was paid to the time frame of the experiment and whether the tests were consistent with the outcome of the systematic review. None of the review authors was blind to the titles of the articles, author names, and affiliations. Statistical significance was based primarily on the interpretation of the *p*-value (*p*-value) for each study individually. Meta-analysis could not be performed due to significant differences in samples. The evidence presented in the studies was based on GRADE (Grading of Recommendations Assessment, Development, and Evaluation) scores for outcomes. For quality assessment of the included studies, researchers used a risk-of-bias tool for preclinical studies RoBDEMAT [18].

## 3. Results

**Figure 1:**
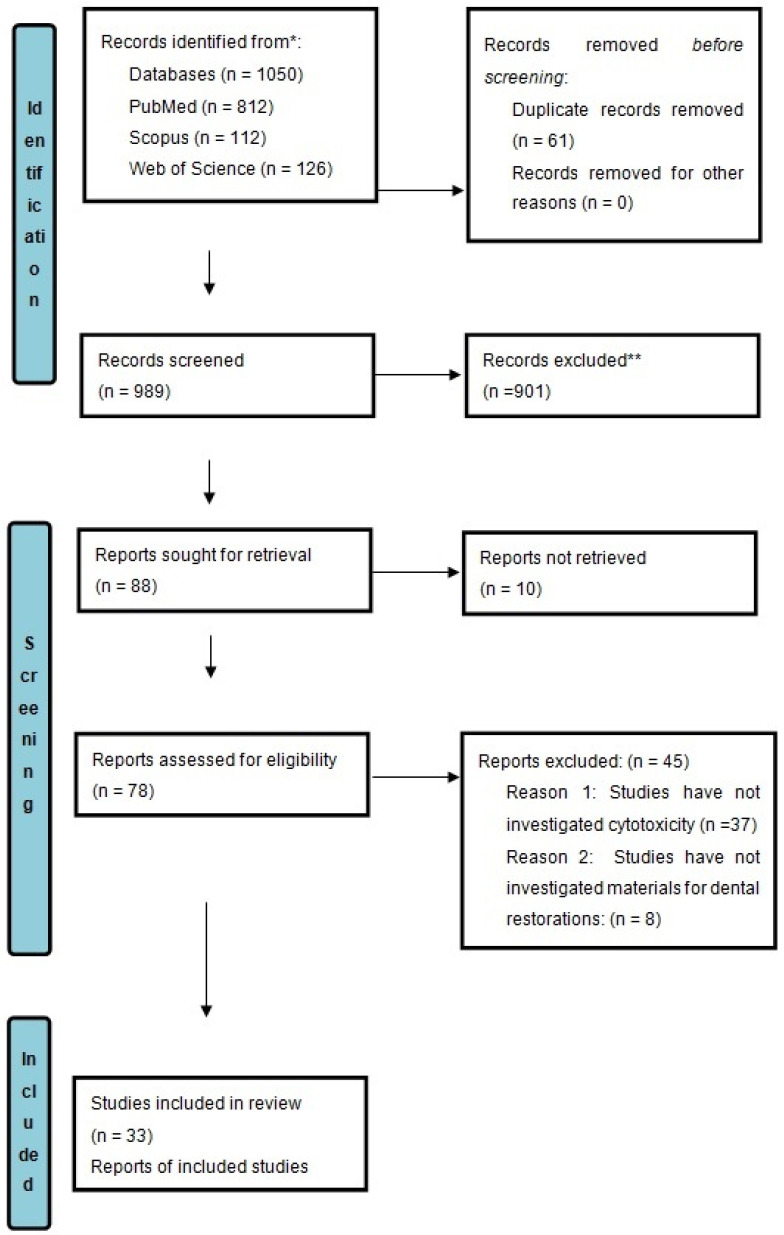
PRISMA 2020 flow diagram of the systematic review protocol used for record retrieval and inclusion. * Reported the number of records identified from each database. ** Records excluded by humans.

Two authors (A.M., J.W.) were involved in the literature identification and screening. The selection process is detailed in the PRISMA 2020 flow diagram in Figure 1. A total of 1050 records were found in the databases: 112 in Scopus, 126 in Web of Science, and 812 in PubMed. Of these records, 989 remained after removing duplicates of studies included in the review. Then, two authors (A.M., J.W.) screened the titles and abstracts of these remaining records based on the inclusion and exclusion criteria, after which 901 articles were excluded. The final evaluation was conducted by two authors (J.W., A.M.), who independently assessed the full text of the 78 selected articles.

Two reviewers (A.M., J.W.) carried out data extraction from chosen studies independently. After data extraction, the third author (J.J.) checked the validity of the retrieved data. The process of data collection included gathering information regarding the types and names of the tested materials, the number of samples, the cell line used, and the outcome significance. The main outcome was to assess the biocompatibility of CAD/CAM milling materials based on the in vitro evaluation of the cytotoxic effects which they induced.

### 3.1. Material Characterization

Table 2 presents the included studies investigating the cytotoxicity of CAD/CAM milled materials on cell lines. In the studies described below, the tested materials came from the same groups with the same processing method but from different manufacturers [19,20,21,22,23,24,25], or were compared to different materials based on milling technique [26,27,28,29,30,31,32,33,34], or were compared to materials processed using classical methods [35,36,37,38,39,40,41,42,43]. Few studies describe experimental materials based on polymers and ceramics [44,45,46,47,48,49]. Scientists describing toxicity used various sources of biological materials. The most common were commercial cell lines of Human Gingival Fibroblasts [20,21,22,25,30,32,34,35,41,47,50] or cells derived from patients [19,26,28,31,33,40,43,44,51]. However, several studies were based on human osteoblasts [37,48], keratinocytes [20,24,30,39,44], dental pulp cells [51], periodontal ligament stem cells [28], or biological material from animals [29,36,38,41,42,44,45,46].

### 3.2. Assessment of Heterogeneity

The issue of statistical analysis heterogeneities primarily stems from the absence of fundamental data, such as the number of samples included in studies, as well as significant variations in study design and methodology. Without detailed sample size information, it becomes challenging to assess the reliability and validity of the findings, as smaller sample sizes can lead to greater variability and reduced statistical power. Furthermore, differing methodologies can introduce biases that affect the comparability of results across studies. This lack of standardization complicates the interpretation of data, making it difficult to draw meaningful conclusions or generalize findings to broader populations. Consequently, the inconsistencies in statistical analyses limit the ability to replicate studies and create a cohesive understanding of the phenomena being examined. The statistical analysis across the reviewed studies exhibits considerable inconsistency. Many papers fail to provide essential details regarding the number of specimens tested, relying instead on averaged data for cell survival and proliferation to draw conclusions [21,22,25,30,41,43,45]. Conversely, some studies employ advanced statistical techniques, utilizing multiple formulas and corrections to enhance analytical sensitivity, including Kolmogorov–Smirnov, Shapiro–Wilk, Levene, and Mann–Whitney U-tests [19,37,44]. The remaining studies base their findings on post hoc analyses along with one-way or two-way ANOVA and Tukey’s test [20,21,23,27,29,34,36,38,39,46].

### 3.3. Risk of Bias

A quality assessment of the selected studies was performed using a risk-of-bias tool, RoBDEMAT [18]. The RoBDEMAT tool incorporates four key domains to assess bias in pre-clinical research systematically. The D1 domain addresses bias in planning and allocation, focusing on control groups, randomization, and sample size determination to ensure robust experimental design. The D2 domain centers on minimizing bias during sample/specimen preparation by standardizing methods. The D3 domain evaluates outcome assessment procedures, ensuring that tests are appropriate for study objectives and free from bias. The D4 domain examines the integrity of data treatment and reporting, emphasizing accurate statistical analysis and comprehensive outcome reporting.

**Figure 2:**
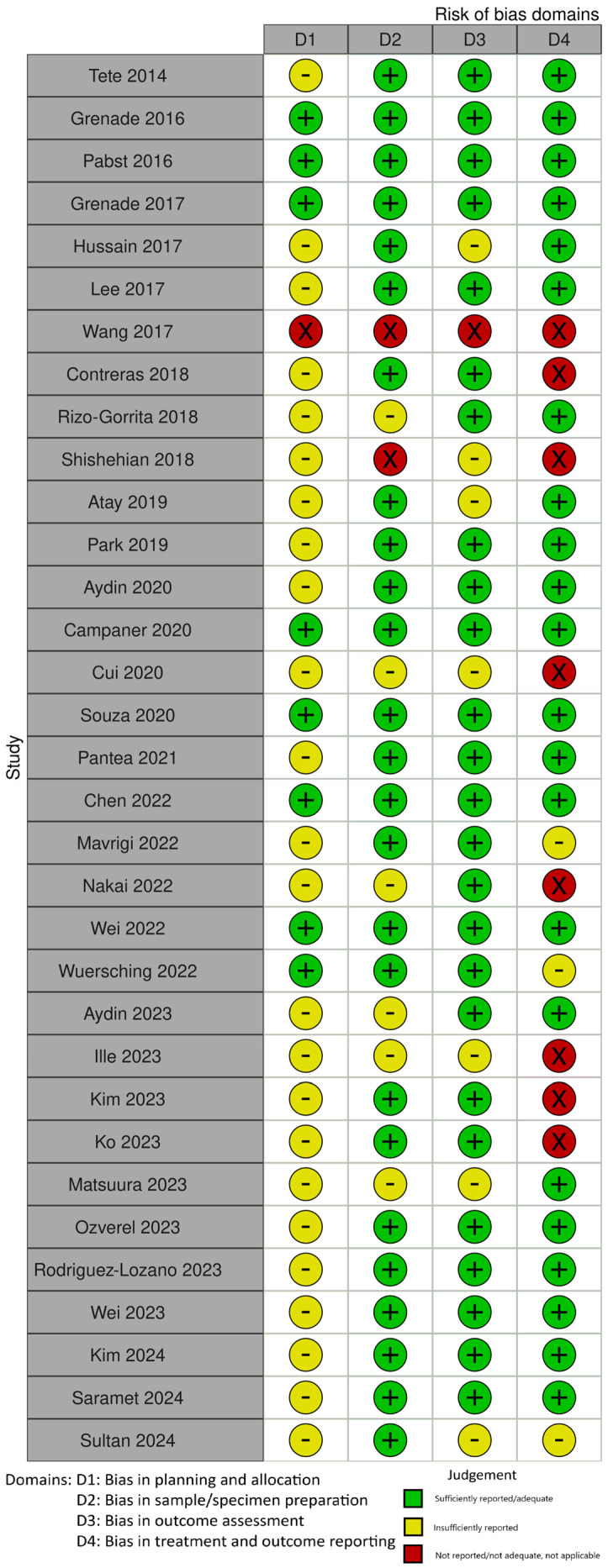
Quality assessment of the included studies using RoBDEMAT in table format [19,20,21,22,23,24,25,26,27,28,29,30,31,32,33,34,35,36,37,38,39,40,41,42,43,44,45,46,47,48,49,50,52].

**Figure 3:**
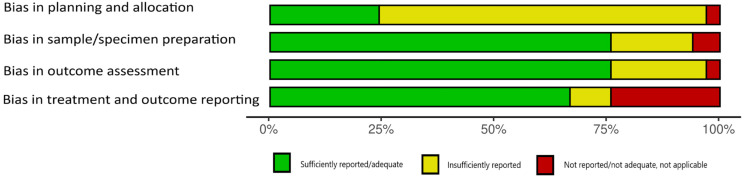
Quality assessment of the included studies using RoBDEMAT in graphical format.

The results of the risk of bias assessment are presented in Figure 2 and Figure 3. Studies were classified as “insufficiently reported” for the following elements: bias in planning and allocation [19,21,22,23,24,25,27,28,29,30,31,32,33,34,35,36,37,41,42,43,46,48,49,52], bias in sample/specimen preparation [22,29,30,32,41,46], bias in outcome assessment [23,25,30,32,35,36,46], and bias in treatment and outcome reporting [25,28,50]. A significant number of papers received a score “not reported, not adequate” in one or more of the four domains due to a lack of information about the sample size, method of preparation, description of any standardization process, presentation of obtained results, or adequate statistical analysis [23,30,31,35,42,46].

### 3.4. Cytotoxicity Evaluation

The researchers performed more than one cell viability test. The cytotoxicity of the materials was most often tested using the MTT assay. This technique was used in fourteen out of thirty-three eligible papers as a validated qualitative method to assess cell metabolic activity [19,21,23,24,25,29,30,33,34,35,38,41,47,49]. The second-most popular toxicity testing method was the MTS, involving four studies out of thirty-three [26,37,44,52]. Some researchers conducted other tests, e.g., the CCK-8 test, WST, Alamar Blue, and immunofluorescence staining. The XTT assay, which provides higher sensitivity and greater dynamic range compared to MTT, was used in only one study conducted by Atay [23]. The method of counting cells using CCK- 8 was reported in six papers [31,40,43,45,46,47]. A fluorescent or confocal microscope was used by many groups to observe the parameters of the nucleus and cytoskeleton [22,26,31,32,37,39,44,48,49,52]. The use of a scanning electron microscope (SEM) has been a very popular imaging method across included articles [19,21,26,28,29,30,39,45,46,48,49]. The enzyme-linked immunosorbent assay (ELISA) as one of the most common tests used in scientific, diagnostic and research studies, has been carried out in only five papers [19,39,40,43,50]. In the context of research into the dental materials cell toxicity, two papers are additionally worth mentioning. The first, presented by Pantea et al., focused on the post-measurement of saliva release of albumin and uric acid as elements significantly affecting toxicity [27]. The second paper, presented by Ille et al. [30], measured, in addition to MTT and LDH assay, the release of nitric oxide, which is related to oxidative stress. Apart from the method of evaluation, significant differences also concerned the duration of experiments, from several hours [19,37,45] to even twenty-one days [28].

### 3.5. Summary of Results

Thirty included studies showed which CAD/CAM-based material had the lowest toxicity to cells. Studies conducted by Hussain et al. [35], Shishehianet al. [23], Ozverel et al. [33], and Sultan et al. [25] showed the toxic effects of different CAD/CAM milled materials. Hussein et al. showed that classical light-cured composite materials have fewer negative effects on cells relative to CAD/CAM resins. According to the author, the cytotoxicity of the tested CAD/CAM material may be caused by the of UV stabilizers, photoinitator, or other particles [35]. Shishehian et al. noted that non-glaze-coated zirconium ceramic exhibit more toxic effects than glazed samples [23]. Ozverel et al. proved that each material tested showed toxicity [33].

### 3.6. Evaluation of the Quality of the Evidence

The quality of the evidence presented in the studies, with overall GRADE (Grading of Recommendations Assessment, Development and Evaluation) scores for outcomes, is shown in Table 3.

The studies reviewed in this study were very heterogeneous; therefore, it was not possible to perform a meta-analysis, and, instead, a narrative and qualitative summary was prepared. The GRADE approach was used to assess the quality of evidence for outcomes [52]. The quality of evidence was assigned to one of the following categories: very low, low, moderate, or high. Articles classified as high-quality evidence were characterized by a detailed description of methodology and obtained results. They included more than one cytotoxicity assay and most often investigated time-distributed cytotoxicity [20,26,38,39,40,44,47,50]. Studies with inaccuracies in methodology, based on a very small sample group, or with a risk of bias, were described as moderate. Studies with low quality of evidence, lacked complete methodological descriptions, lacked basic information about the size of the study group, or presented a risk of bias were described as low. None of the studies included were classified as very low quality of evidence.

## 4. Discussion

This systematic review focused primarily on the question: are modern CAD/CAM milled materials biocompatible? The initial assessment of biocompatibility was based on conducting in vitro cytotoxic studies by performing indirect or direct tests aimed at evaluating cytotoxic effects on cells in order to determine the safety and functionality of materials used in dental applications [27,33,49].

Authors have often focused on a selected aspect under research, thus limiting the holistic approach to the topic by strictly attempting to explore one of many biocompatibility aspects. Shishehian et al. and Rizo-Gorrita et al. paid significant attention to the roughness of the surfaces of the designed reconstructions. They noted that significant roughness of zirconium restoration is highly prone to bacterial colonization and will lead to increased biofilm deposition, directly affecting the cells’ immune response [22,23]. Rizo-Gorrita highlighted the need to reconsider how roughness parameters are utilized in in vitro studies [22]. It was pointed out that the arithmetic roughness value (Ra) is a limited measure for complex topographies. In this research, surface roughness was measured using profilometric testing based on ISO 25178 [53], whereas many studies that examine cellular responses in relation to roughness primarily report arithmetic roughness values.

Most of the authors of the reviewed papers limited themselves to one cell line when designing the study [19,20,21,22,23,24,25,28,31,32,33,34,36,37,38,39,40,42,43,46,47,48,49,50]. The duration of biological load averaged up to 72 h, with the longest study lasting 21 days (Mavriqi et al.), significantly limiting the long-term clinical implications [28], assuming that fixed prosthetic restorations are expected to function in the oral cavity for many years [33].

Ille et al. and Pabst et al. conducted studies in which reconstruction materials were tested using two cell lines, Human Gingival Fibroblasts (HGFs) and Human Gingival Keratinocytes (HGKs) [20,30]. Regardless of the materials tested, all showed significantly greater toxicity and proliferation disorders concerning HGKs than HGFs. In the context of broad biocompatibility, Park et al. commented on testing using pre-osteoblast cells, highly relevant to the oral environment, especially in implantology [37], while Maviqi et al. used human periodontal ligament stem cells [28]. These are the only studies where researchers pay significant attention to the use of appropriate cell lines for a particular type of dental reconstruction, which adds valuable data for determining possible cytotoxic effects [28,37]. Pantea, Ozverel, and Kim conducted their studies using both direct (material in contact with cells) and indirect (extract of material in contact with cells) tests [27,33,49]. A significant proportion of collected studies for this review were based on direct assays in which cell lines were cultured on previously prepared material samples [19,20,21,22,23,24,25,26,28,29,30,31,32,34,36,37,38,39,40,41,42,43,44,45,46,47,48,50]. It is worth noting that the cytotoxicity tests over time were conducted in most of the studies, but some groups evaluated cellular effects at only one time point [22,27,28,29,30,31,39,40,42,52]. This is important because it shows that long-term side effects of monomer release by polymer materials were not always thoroughly investigated. On the other hand, the degradation behavior of dental materials and releasing of potentially harmful particles were evaluated by two research groups, using artificial acidic saliva [30] and human saliva [27]. Factors such as exposure to saliva, enzymes, rapid temperature changes, pH variations, and mechanical stresses can influence the degradation process.

The in vitro studies conducted by Grenade, Wang, and Kim revealed that experimental polymer-infiltrated ceramic network (PICN) materials show promise as a new generation of CAD/CAM restoratives [44,45,49]. According to Wang, no monomer release from PICN was detected, nor was any indirect cytotoxicity observed. The improved mechanical properties of this material, coupled with good biocompatibility, could make it a suitable alternative to existing materials. According to the author, further research is necessary to evaluate its long-term performance, clinical handling characteristics, and potential for wear. It can be presumed that the study conducted by Grenade et al. even better mimicked the true toxicity effects because of the very long exposure time. Lithium disilicate and V-grade titanium, compared to hybrid polymer–ceramic studied by Grenade et al., gave intermediate results of cell proliferation, especially for implant abutments, which were examined from this point of view [45].

Very extensive studies describing temporary reconstruction materials were conducted by Wei and Wursching [40,50]. They compared conventional materials to those processed by CAD/CAM. Both studies showed trace cytotoxicity of CAD/CAM polymers, indicating good industrial polymerization technology. Campaner et al., Souza et al., and Kowith et al. focused on a narrow group of the most commonly used materials [38,39,42]. Noteworthy were the different cell lines used, such as oral epithelial cells and human gingival fibroblasts. Testing materials on different cell lines presents a wider range of effects of residual monomers on varied oral cells.

Ceramic materials are considered highly biocompatible materials due to their chemical inertness. Zirconia-reinforced lithium silicate (ZLS) is an innovative dental material with a unique chemical composition that is designed to combine harmoniously with the appropriate optical properties of lithium disilicate and the enhanced mechanical strength of zirconia. Much doubt still exists regarding the biocompatibility of ZLS ceramic. While zirconia has no toxic effects on bone tissue, there have been no studies on how ZLS affects human gingival keratinocytes and fibroblasts. It was not until the Rizo-Gorrita study that any doubts were dispelled, and statistically lower cytotoxicity of Y-TZP compared to ZLS was confirmed. A parameter often overlooked is the effect of crystallization of lithium disilicate-based ceramic materials. The Mavriqi et al. study of crystallization parameters clearly indicated the need to control them very precisely. When crystallization was carried out according to the assumed parameters, the cytotoxicity of lithium disilicate was significantly lowered.

Over the last ten years, there have been significant advancements in dental materials, as well as many studies on their toxicity. The diversity of available products significantly complicates the choice of a safe and durable solution for the patient. According to the systematic review above, all materials processed by CAD/CAM milling technology are either significantly less toxic or slightly less toxic compared to conventional techniques using analogous materials. The choice of a particular material dictated by the clinical situation remains an issue. It is worth noting that in this systematic review, the literature reviewed was usually focused on one narrow section of research. Each of the reviewed studies has very strong points showing small but very important aspects of knowledge about the dental materials used.

Elaborating on the limitations in current research on dental materials reveals several critical areas for improvement. Variability in test conditions, such as differences in cell lines, assay types, and exposure times, can lead to inconsistent results that complicate the interpretation of biocompatibility. Furthermore, the lack of standardization in surface treatments of materials can affect their properties and interactions with biological tissues, making comparisons across studies challenging. The absence of long-term degradation studies and microbiological interaction assessments is another significant limitation. These factors are essential for understanding how materials behave in the complex oral environment over time. Future studies should incorporate multi-cell line assays to capture a broader spectrum of biological responses. Additionally, utilizing real-time cytotoxicity platforms could provide more immediate insights into the effects of dental materials. Simulating oral microenvironment factors, such as temperature cycling and enzymatic activity, would enhance the relevance of laboratory findings to clinical practice.

It is worth mentioning that surface finishing, aging simulations, and ion release in acidic oral environments are critical factors that can significantly influence the long-term biocompatibility of dental restoration materials [23,31,46,47]. These simulations help predict how dental materials will behave over time, revealing their potential for wear and chemical degradation. Ion release studies are pivotal in assessing how materials respond to acidic environments, which can lead to the leaching of metal ions, potentially causing adverse biological reactions [31]. Such extensive studies are extremely difficult to conduct. The synergistic effects of mechanical wear and chemical degradation are often overlooked, and only a small number of papers present a broad view on examined dental materials [31]. Understanding how materials degrade over time helps predict their potential risks associated with degradation byproducts [15,16]. However, this issue was not in the scope of the current review; a more detailed analysis of the studies focused on this topic could be performed in the future.

The main limitation of this review is the lack of a meta-analysis, which could not be performed due to the heterogeneity of the available reports. The use of different cell lines from animals and humans made it impossible to conduct a reliable analysis, so results were compared only within studies but not between studies. Furthermore, some of the reports did not precisely define the full names of the commercial or experimental materials used. The identified risk of bias can be attributed mainly to the lack of information regarding the number of operators performing the experiments, low sample size, and control groups strongly different from tested samples, which were observed in several studies. On the other hand, a significant advantage of this systematic review is the selection of papers describing studies that compare modern, widely used CAD/CAM dental restoration materials and experimental ones.

## 5. Conclusions

Depending on the material tested, those milled in the CAD-CAM system showed no or very limited toxicity compared to conventional ones for both ceramic and polymer materials. This effect appears to be closely correlated with the processing technology of the mentioned materials. The blocks of polymeric materials are processed using high pressure and temperature, reducing the amount of monomer, but not eliminating it completely.

## Figures and Tables

**Table 1:** Inclusion and exclusion criteria.

PICOS	Inclusion Criteria	Exclusion Criteria
**Population**	Milled materials for fixed dental restoration	3D-printed materials Samples used for filling restoration Materials for denture base plates
**Intervention**	Incubation with in vitro cell cultures (direct or non-direct contact tests)	Lack of any biological study
**Comparator**	Conventional (not milled) or 3D-printed materials	None
**Outcome**	Assessing the cytotoxicity of CAD/CAM dental milled materials	Only material properties evaluation
**Study**	Only English language research articles published between 1 January 2014 and 31 October 2024	Review articles Articles published before 1 January 2014 Articles not in English

**Table 2:** Characteristics of the materials and cells used in studies included in this systematic review, as well as the main outcomes of the research, presented in chronological order.

Author and Year	CAD/CAM Material	Sample Size	Cell Line Name	Biocompatibility Test	Experiment Time Frame	Primary Outcome	Side Effect
Tete 2014 [19]	1. **IPS e.maxZirCAD** (IvoclarVivadent AG, Schaan, Liechtenstein), with no surface treatment 2. **IPS e.maxZirCAD** (IvoclarVivadent, Schaan, Liechtenstein), ground and polished 3. **IPS e.max** (Ivoclar Vivadent, Schaan, Liechtenstein) ground and polished 4. **feldspathic veneering ceramic** (conventional control, non-CAD-CAM)	n = 144	**Human gingival fibroblasts** (non-commercial)	- MTT assay - ELISA (type I collagen secretion) - LDH assay - SEM analysis	- 3 h - 24 h - 72 h	More positive cell response in contact with ground and polished zirconia than with lithium disilicate or feldspathic ceramic was revealed.	Lower fibroblast growth rate and collagen I secretion when culturing cells on conventional feldspathic ceramic and IPS e.max was revealed.
Grenade 2016 [26]	1. **Experimental Polymer-infiltrated ceramic network—PICN** 2. **Grade V titanium** (Procera, Nobel Biocare, Kloten, Switzerland) 3. **Y-TZP** (Procera, Nobel Biocare, Kloten, Switzerland) 4. **IPS e.max Press** (Ivoclar Vivadent, Schaan, Liechtenstein)	n = 136	**Human gingival fibroblasts** (non-commercial)	- MTS assay - Immunofluorescence staining of actin filaments and nuclei - SEM analysis	- 24 h - 48 h - 72 h	Titanium and zirconia were most biocompatible; PICN exhibited comparable results to lithium disilicate glass-ceramic.	Experimental Polymer-infiltrated ceramic network—PICN—showed good cellular attachments but slower proliferation than titanium and zirconia.
Pabst 2016 [20]	1. **Sirona inCoris ZI** (Dentsply Sirona, Bensheim, Germany) 2. **VITA In-Ceram YZ** (VITA Zahnfabrik, Bad Säckingen, Germany) 3. **Ivoclar IPS e.maxZirCAD** (Ivoclar Vivadent, Schaan, Liechtenstein)	n = 160	**Human gingival fibroblasts** (Lonza, Basel, Switzerland) **Human oral keratinocytes (HOK)** (Provitro, Heidelberg, Germany)	- MTT assay - Scratch assay - ToxiLight assay (ADK release)	- 3 days - 6 days - 9 days - 12 days	The viability and migration ability of HOKs were negatively influenced by the tested CAD/CAM zirconia ceramics, whereas their biocompatibility with HGFs was better.	Lower viability and migration ability of oral keratinocytes on all tested zirconium-based materials.
Grenade 2017 [44]	1. **Experimental Polymer-infiltrated ceramic network—PICN** 2. **grade V titanium** (Procera, Nobel Biocare, Kloten, Switzerland) 3. **Y-TZP** (Procera, Nobel Biocare, Kloten, Switzerland) 4. **IPS e.max Press** (Ivoclar Vivadent, Schaan, Liechtenstein) 5. **Polytetrafluoroethy-lene** (Sirris, Seraing, Belgium) as negative control	n = 182	**Human gingival keratinocytes** (non-commercial) and L929 mouse fibroblasts	- MTS assay - Immunofluorescence staining (vinculin, actin, nuclei)	- 24 h - 48 h - 72 h	Results similar to HGFs [20], but slightly better biocompatibility of PICN to HGKs compared with HGFs.	Slightly lower HGF cell adhesion to PICN when compared with HGK adhesion.
Hussain 2017 [35]	1. **Lava Ultimate** (3M ESPE, Seefeld, Germany) 2. **Vita Enamic** (VITA Zahnfabrik, Bad Säckingen, Germany) 3. **Paradigm MZ100** 4. **Filtek Z250** (3M ESPE, Seefeld, Germany) 5. **Tetric EvoCeram**(Ivoclar Vivadent, Schaan, Liechteinstein)	n = 50	1. **Epithelial lung carcinoma cell line** (A549, ATCC, Manassas, VA, USA) 2. **Human gingival fibroblasts** (Provitro GmbH, Heidelberg, Germany)	- LDH assay - Cell morphology	- 24 h - 48 h	Cytotoxicity of LAVA, VITA, andMZ100 was above the maximum value accepted for cytotoxicity of medical devices according to ISO-10993:5 [51].	Extracts from LAVA Ultimate, VITA Enamic, andParadigm MZ100 showed high LDH activity (above the maximum value accepted in ISO 10993:5).
Lee 2017 [52]	1. **Snap** (Parkell, Edgewood, NY, USA) 2. **Jet** (Lang Dental, Rochester, NY, USA) 3. **Luxatemp** (DMG, Hamburg, Germany) 4. **Revotec LC** (GC, Luzern, Switzerland) 5. **Vipi block** (Vipi, Pirassununga-SP, Brazil)	n = 15	**Primary cultured human dental pulp cells** -hDPCs(non-commercial)	- MTS assay - Cytokine expression analysis - Confocal laser scanning microscopy (live/dead assay)	- 24 h	CAD/CAM material (Vipi block) achieved better biocompatibility compared to conventional resin (Snap, Jet, Luxatemp).	Extracts from the conventional resins (Snap, Jet, Luxatemp), especially in the initial polymerizing state, significantly lowered the cell viability without inducing proinflammatory cytokines. CAD/CAM resin blocks (Vipi block) were not cytotoxic.
Wang 2017 [45]	1. **Experimental PICN** **Composites**	No data	**Rat bone mesenchymal stem cells** (source not defined)	- CCK 8 assay	- 1 day - 3 days - 5 days	The fabricated PICN showed good biocompatibility.	No side effects detected.
Contreras 2018 [21]	1. **Vita Mark II blocks** (Vita Zahnfabrik, Bad Säckingen, Germany) **2. Vita VM9 stratified ceramic powder and modeling liquid** (Vita Zahnfabrik, Bad Säckingen, Germany)	No data	**Human gingival fibroblasts FMM-1 cell line** (source not defined)	- MTT assay - SEM	- 24 h - 7 days	No cytotoxicity detected	No side effects detected
Rizo-Gorrita 2018 [22]	1. **Vita YZ** (Vita Zahnfabrik, Bad Säckingen, Germany) 2. **Celtra Duo** (Degudent, Hanau-Wolfgang, Germany)	No data	**Human gingival fibroblasts** (Lonza, Basel, Switzerland)	- Nuclear and cytoskeletalparameters measured using confocal microscopy	- 24 h	Lower cytotoxicity of Y-TZP Vita compared to ZLS Celtra Duo.	HGFs cultured on ZLS Celtra Duo showed lower spreading, proliferation, and coverage than cells cultured on Y-TZP (Vita YZ T).
Shishehian 2018 [23]	**Ceramill Zolid FX** (Amann Girrbach AG, Maeder, Austria): 1. glazed chemically 2. polished 3. glazed by laser radiation 4. intact	n = 20	**Fibroblasts** (source not defined)	- MTT assay	- 72 h - 1 week	Polished only zirconium restorations can result in incompatible cellular response.	Glazing of zirconia improved its biocompatibility; intact samples had inhibitory effect on mitochondrial SDH activity in cells, while the glazed group revealed the least inhibitory response.
Atay 2019 [36]	1. **Lava Ultimate** (3M ESPE, Seefeld, Germany) 2. **VITA Mark II** (Vita Zahnfabrik, Bad Säckingen, Germany) 3. **InCoris TZI** (Ivoclar Vivadent, Schaan, Liechteinstein) 4. **IPS e.max CAD** (Ivoclar Vivadent, Schaan, Liechteinstein) 5. **VITA Suprinity** (Vita Zahnfabrik, Bad Säckingen, Germany) 6. **Cerasmart** (GC, Luzern, Switzerland) 7. **IPS Empress CAD** (Ivoclar Vivadent, Schaan, Liechteinstein) 8. **Protemp**4 (3M ESPE, Seefeld, Germany) 9. **Telio CAD** (Ivoclar Vivadent, Schaan, Liechteinstein) 10. **CAD-Temp** 11. **Telio Lab** (Ivoclar Vivadent, Schaan, Liechteinstein) 12. **Temdent Classic** (Schutz Dental, Rosbach, Germany) 13. **Telio CS C&B** (Ivoclar Vivadent, Schaan, Liechteinstein)	n = 312	**L929 Mouse fibroblast cell line** (ATCC, Manassas, VA, USA)	- XTT assay - Apoptosis determined by Annexin-V/PI staining and analyzed by flow cytometry	- 1 day - 3 days - 7 days	CAD/CAM ceramics and polymers showed very low cytotoxicity.	Leucite-based glass ceramic material (IPS Empress CAD) showed the lowest cell viability and the highest apoptosis rate among all-ceramic materials on the 7th day. The resin matrix ceramics showed favorable viability results.
Park 2019 [37]	1. **ZenostarT** (Wieland Dental, Pforzheim, Germany) 2. **Co-Cr** (StarLoy C, Degudent, Hanau-Wolfgang, Germany) 3. **Ni-Cr** (VeraBond 2V, Aalba Dent, Fairfield, CA, USA) 4. IPS e.max Press (Ivoclar Vivadent, Schaan, Liechteinstein) 4. **Porcelain fused to Gold** (P.F.G.) (Myeso X Yesbiogold, Republic of Korea)	n = 4	**Pre-osteoblast cell line** (MC3T3-E1 ATCC, Manassas, VA, USA)	- CLSM (actin and nuclei) - MTS assay - ALP activity assay	- 6 h - 24 h - 5 days - 14 days	Sufficient cytocompatibility of P.F.G, IPS e.max Press, ZenostarT and Co-Cr	Cells were not able to spread out on Ni-Cr (VeraBond 2V). Cell adhesion, proliferation, and differentiation were better in dental ceramic materials and Co-Cr.
Aydin 2020 [24]	1. **Vita Enamic** (Vita Zahnfabrik, Bad Säckingen, Germany) 2. **Cerasmart** (GC, Luzern, Switzerland) 3. **Grandio Blocs** (VOCO, Cuxhaven, Germany) 4. **Brilliant Crios** (Coltene, Altstatten, Switzerland) 5. **Celtra Dou** (Dentsply Sirona, Bensheim, Germany)	n = 4	**Human gingival keratinocytes** (source not defined)	- MTT assay	- 1 day - 3 days - 7 days	Vita Enamic prepared by the PINC method showed the lowest cell toxicity. All the CAD/CAM blocks showed cell viability above the level acceptable by ISO (70%).	ZLS (Celtra Dou) showed the lowest cell viability. All CAD/CAM blocks showed cell viability above the acceptable level (70%).
Campaner 2020 [38]	1. **HPAR- acrylic resin** (Vipi, Pirassununga-SP, Brazil) 2. **APR** (Alike, Reliance Dental Mfg CO, Worth, IL, USA) 3. **Protemp 4** (3M ESPE, Seefeld, Germany) 4. **Lava Ultimate** (3M ESPE, Seefeld, Germany) 5. **Telio CAD** (Ivoclar Vivadent, Schaan, Liechtenstein)	n = 120	**Mouse gingival fibroblasts** (non-commercial)	- MTT assay - Alamar Blue assay - ELISA (levels of cytokines)	- 24 h - 48 h - 72 h	CAD/CAM materials were less cytotoxic than auto-polymerized acrylic resin (Alike) and bisacrylic resin (Protemp 4).	Acrylic resin (Alike) and bisacrylic resin (Protemp 4) showed the lowest cell viability as well as increased IL-6, IL-1β and TNF-α levels.
Cui 2020 [46]	**Experimental PICN** Concentrations of 1, 2, 3, and 4 wt% (referring to nano- hydroxyapatite powders)	n = 24	Rat bone marrow mesenchymal stem cells (rBMSCs) (source not specified)	- CCK-8 assay - SEM	- 1 day - 3 days - 5 days	The bio- compatibility of PICNs can be improved by the addition of hydroxyapatite nano-powders.	PICNs without hydroxyapatite show poor cell proliferation The addition of hydroxy-apatite can improve the proliferation and attachment of rBMSCs for both porous ceramics and PICNs.
Souza 2020 [39]	1. **Dencôr** (Dental Articles LTDA) 2. **Protemp 4** (3M ESPE, Seefeld, Germany) 3. **Vipiblock** (Vipi, Pirassununga-SP, Brazil)	n = 48	Normal oral keratinocites (NOK—SI cell line) (source not specified)	- Alamar Blue assay - LIVE/DEAD assay - ELISA (epidermal growth factor synthesis) - Immunofluorescence staining (actin and nuclei) - SEM	- 24 h	CAD-CAM-type resin (Vipi block) showed the lowest cytotoxicity.	Conventional acrylic resin (Dencôr color 66) and bis-acrylic resin (Protemp 4) showed lower cell viability and adhesion, as well as epidermal growth factor (EGF) synthesis, than CAD/CAM-type resin.
Pantea 2021 [27]	1. **Telio CAD** (Ivoclar Vivadent AG, Schaan, Liechtenstein) 2. **NextDent C&B MFH** (3D Systems, Soesterberg, The Netherlands) 3. SR **Chromasit** (Ivoclar Vivadent AG, Schaan, Liechtenstein) 4. Superpont C+B (Spofa Dental, KaVo Kerr Group, Jicin, Czech Republic)	n = 12	**Saliva samples**	- Uric acid, GGT, OXSR-1, TAC, TNFα and IL-6 measured using analyzing kits	- 12 h	The obtained biochemical data showed that the tested materials did not significantly modify the antioxidant capacity of the incubated saliva as well as the salivary inflammatory status.	No side effects detected.
Chen 2022 [47]	**Hybrid Polymer–Ceramic Material** (non-commercial)	n = 12	**Human gingival fibroblast** (Wuhan Biotower Biotechnology, Wuhan, China)	- MTT assay immunofluorescence staining (cytoskeleton and nuclei)	- 48 h - 72 h	Short-term exposure of human gingival fibroblasts to the hybrid polymer–ceramic material does not cause cytotoxicity.	No side effects detected.
Mavriqi 2022 [28]	1. **IPS EMax-CAD** (Ivoclar Vivadent, Schaan, Lichtenstein) 2. **Vita Suprinity PC** (VITA Zahnfabrik, Bad Säckingen, Germany) 3. **Celtra Duo** (Dentsply Sirona, Bensheim, Germany)	n = 3	**Human periodontal ligament stem cells**—hPDLSC (non-commercial)	- SEM	- 21 days	Lower cytotoxicity if CAD/CAM materials are properly crystallized. Better biocompatibility of lithium disilicate (IPS e.max CAD) than two zirconia reinforced lithium silicates.	Human periodontal ligament stem cells better adhere onto CAD/CAM lithium disilicate than onto ZLS surfaces.
Nakai 2022 [29]	1. **Katana Avencia Block** (Kuraray Noritake Dental, Tokyo, Japan) 2. **Shofu Block** (Shofu, Kyoto, Japan) 3. **Artesano** (Yamahachi Dental, Gamagori, Japan) 4. **VITABLOCS Mark II** (VITA Zahnfabrik, Bad Säckingen, Germany) 5. **IPS Empress CAD** (Ivoclar Vivadent, Schaan, Lichtenstein)	n = 15	**Mouse-derived fibroblast-like cells (Balb/c 3T3 cells)** (Riken BioResource Research Center, Ibaraki, Japan) **fetal rat skin-derived keratinocytes (FRSK cells)** (JCRB cell bank (Osaka, Japan))	- Oxidative stress (ROS assay) - MTT assay - SEM - Apoptosis determined by Annexin-V/PI staining and analyzed by flow cytometry - Hematoxylin and eosin staining	- 24 h	Slightly more toxic resin-based materials (Katana, Shofu Block, Artesano) compared to ceramic (Vitablocks Mark II, IPS EmpressCAD).	Resin materials were more toxic than ceramics.
Wei 2022 [40]	1. **Vertex**acrylic resin (Vertex, Soesterberg, The Netherlands) 2. **Organic PMMA** (Organical CAD/CAM, Berlin, Germany) 3. **Protemp 4** (3M ESPE, Seefeld, Germany) 4. **Aidite CAD/CAM polymer** (Aidite Technology Co., Qinhuangdao, Hebei, China)	n = 540	**Human gingival fibroblasts** (non-commercial)	- CCK-8 - Apoptosis determined by Annexin-V/PI staining and analyzed by flow cytometry - Apoptosis-related gene expression (RT-qPCR) - Apoptosis-related protein expression (Western blot) - Levels of inflammatory cytokines (ELISA)	- 72 h	CAD/CAM dental polymers (Organic PMMA, Aidite CAD/CAM) have favorable biocompatibility.	The biocompatibility of CAD/CAM polymers was significantly better than conventional polymers. Conventional bis-acrylic composite resin (Protemp 4) can affect cell proliferation through the intrinsic mitochondrial apoptosis.
Wuersching 2022 [50]	1. **VarseoSmile Crown plus** (BEGO, Bremen, Germany) 2. **NextDent** C&B MFH (NextDent, Soesterberg, The Netherlands) 3. **VarseoSmile Temp** (BEGO, Bremen, Germany) 4. **Temp PRINT** (GC, Luzern, Switzerland) 5. **Tetric CAD** (Ivoclar Vivadent, Schaan, Lichtenstein) 6. **Telio CAD** (Ivoclar Vivadent, Schaan, Lichtenstein) 7. **Tetric EvoCeram** (Ivoclar Vivadent, Schaan, Lichtenstein) 8. **Protemp 4** (3M ESPE, Seefeld, Germany) 9. **P Pro Crown & Bridge** (Straumann, Basel, Switzerland)	n = 135	**Human gingival fibroblast hGF-1** (LGC Standards, Wesel, Germany)	- Cell viability (RealTime-Glo^®^ MT Cell Viability Assay) - Inflammatory response -levels of IL-6 and PGE_2_ (ELISA) - Oxidative stress (GSH/GSSG-Glo™ Assay - Induction of apoptosis (RealTime-Glo™ Annexin V Apoptosis and Necrosis Assay)	- 24 h - 72 h	Tetric CAD and Telio CAD were slightly toxic. All other resins were moderately to severely cytotoxic.	VarseoSmile Crown plus and P Pro Crown & Bridge significantly enhanced PGE2 levels. Higher concentrations of oxidized gluthatione were determined in the presence of Telio CAD, VarseoSmile Temp, and P Pro Crown & Bridge. All printable resins slightly induced apoptosis.
Aydin 2023 [41]	1. **Crowntec** (Saremco Dental AG, Rebstein, Switzerland) 2. **Permanent Crown** (Formlabs, Somerville, MA, USA) 3. **Vita Enamic** (VITA Zahnfabrik, Bad Säckingen, Germany) 4. **Brilliant Crios** (Crios, Coltene, Altstatten, Switzerland) 5. **Clearfill Majesty Posterior** (Clearfil Kururay, Tokyo, Japan)	No data	**L-929 mouse fibroblasts** (source not specified) **Human gingival fibroblast** (HGF-1, ATCC, Manassas, VA, USA)	- MTT assay	- 24 h - 72 h	3D-printed permanent restoration resins (Crowntec and Permanent Crown) showed similar cell viability on HGF-1 and L929 cells to resin-based CAD/CAM blocks (Vita Enamic, Brilliant Crios) and composite resin (Clearfill Majesty Posterior),	No significant side effects detected.
Ille 2023 [30]	1. **Cerasmart** (GC, Luzern, Switzerland) 2. **Straumann Nice** (Nice Straumann, Freiburg, Germany) 3. **TetricCAD** (Ivoclar Vivadent, Schaan, Liechtenstein)	No data	**Normal human fibroblasts** (BJ cell line, ATCC, Manassas, VA, USA) **Normal human keratinocytes** (HaCaT cell line, CLS—Cell Line Services)	- MTT assay - LDH assay - Nitric oxide production (Griess assay) - SEM	- 24 h	CAD/CAM (Cerasmart, Straumann Nice, Tetric CAD) restorative materials tested are biocompatible,	The restorative materials tested ranged from moderately cytotoxic to slightly cytotoxic to noncytotoxic on the growth of human fibroblasts. The samples tested can be considered slightly cytotoxic and noncytotoxic, with the exception of SN_B (Straumann Nice), which recorded a reduced mitochondrial activity of keratinocytes.
Kim 2023 [31]	1. **IPS e.max CAD** (Ivoclar Vivadent, Schaan, Liechtenstein) 2. **Celtra Duo** (Dentsply Sirona, Bensheim, Germany) 3. **Vita Enamic** (VITA Zahnfabrik, Bad Säckingen, Germany) 4. **Cerasmart** (GC, Luzern, Switzerland) 5. **Lava Plus Zirconia** (3M ESPE, Seefeld, Germany)	n = 225	**Human gingival fibroblasts** (non-commercial)	- LIVE/DEAD assay (fluorescence microscope) - CCK-8 assay - Immunocytochemistry of cell spreading (staining of actin and nuclei) and focal adhesion (Talin 1 and vinculin)	- 24 h	Zirconia-reinforced lithium silicate (Celtra Duo) exhibited significantly lower cell viability compared to other materials.	Zirconia-reinforced lithium silicate (Celtra Duo) exhibited significantly lower cell viability compared to other materials.
Ko 2023 [42]	1. **Temp Basic 95H16**, (Zirkonzahn, Gais, Italy) 2. **Vertex Self Curing** (Vertex, Soesterberg, The Netherlands)	n = 10	**L929 fibroblast cell line** (source not defined)	WST assay	- 24 h	Both hand-mixed self-curing resin and CAD/CAM polymer (Temp Basic) showed low cytotoxicity	No side effects detected.
Matsuura 2023 [32]	1. **Aeliteflo** (BISCO, Schaumburg, IL, USA) 2. **Aelite Aesthetic Enamel** (BISCO, Schaumburg, IL, USA) 3. **Vivid PMMA Disc Pearson** (Dental Supply Co., Sylmar, CA, USA)	n = 120	**Human gingival fibroblasts** (ScienCell Research Laboratories, Carlsbad, CA, USA)	- Fluorescence microscopy (staining of actin and nuclei) - Collagen production (Picrosirius-red staining)	- 2 days - 4 days - 6 days	CAD/CAM polymer (Vivid PMMA Disc Pearson) showed lower cytotoxicity.	No cells survived and attached to or around the flowable composite (Aeliteflo™), regardless of cure time. No cells attached to the bulk-fill composite (Aelite™ Aesthetic Enamel), but some cells survived around the material, and the number increased with longer cure times. Milled acrylic (Vivid PMMA) was the most biocompatible.
Ozverel 2023 [33]	1. **IPS e.max CAD** (Ivoclar Vivadent, Schaan, Liechteinstein) 2. **LavaUltimate** (3M ESPE Seefeld Germany) 3. **TetricCAD** (Ivoclar Vivadent, Schaan, Liechteinstein)	n = 432	**Human embryonic kidney epithelial cells** HEK293 (source not defined)	- MTT assay	- 5 days - 10 days - 15 days	All restorative materials decreased the viability of cells.	All the restorative materials decreased the viability of cells.
Rodríguez-Lozano 2023 [48]	1. **Milled yttria-stabilized tetragonal zirconia polycrystal disks** (Priti multidisc ZrO 2monochrome; Pritidenta) 2. Y-TZP zirconia material (LithaCon 3Y 210; Lithoz, Troy, NY, USA) for lithography-based ceramic additive manufacturing	n = 20	Normal human osteoblasts (NHOsts; Lonza, Basel, Switzerland	- Resazurin-based viability assay - Wound healing assay - Confocal microscopy (immunofluorescence staining of actin and nuclei) - SEM	- 24 h - 48 h - 72 h	Lithography-based zirconia showed similar cytocompatibility when compared with the milled zirconia.	No side effects were detected. Lithography-based zirconia showed similar cytocompatibility when compared with the milled zirconia.
Wei 2023 [43]	1. **BioHPP CAD/CAM** (Bredent, Senden, Germany) 2. **Conventional bioHPP** (Bredent, Senden, Germany)	No data	**Human gingival fibroblasts** (non-commercial)	- CCK-8 assay - Apoptosis assay determined by AnnexinV-FITC/PI kit and analyzed by flow cytometry - Apoptosis-related gene expression (RT-qPCR) - Apoptosis-related protein expression (Western blot) - Levels of IL-6, IL-1β, and TNF-α (ELISA)	- 24 h - 48 h - 72 h	Fabrication method did not affect the biological properties of modified PEEK; both press and CAD/CAM milled materials were biocompatible.	Culturing of HGFs with the eluates from both materials increased the mRNA expression levels of Bax and Caspase-3 and downlegulated the level of Bcl-2 gene expression.
Kim 2024 [49]	1. **Experimental Nano-crystalline ceramic and polymer (NCP)** with 2-methacryloyloxyethyl phosphorylcholine (MPC) and sulfobetaine methacrylate (SBMA)(MS) 0.15 wt% 2. **Experimental Nano-crystalline ceramic and polymer** with 2-methacryloyloxyethyl phosphorylcholine (MPC) and sulfobetaine methacrylate (SBMA) 0.45 wt%	n = 13	**Human gingival fibroblasts HGF-1** (source not specified)	- MTT assay - Anaerobic bacterial attachment resistance analyses (SEM) - Live/dead bacterial staining (CLSM)	- 24 h - 48 h - 72 h	NCP containing 0.15% MS can effectively reduce adhesion of bacteria and shows low cytotoxicity.	No side effects detected.
Saramet 2024 [34]	1. **Dental Sand** (HARZLabs LLC, Moscow, Russian Federation) resin for 3D printing 2. **HUGE dental PMMA blocks** (MedNet EC-REP, Munster, Germany)	n = 50	**Human gingival fibroblasts HGF-1** (American Type Culture Collection ATCC, Manassas, VA, USA)	- MTT assay - Nitric oxide level (Griess assay) - LDH assay - LIVE/DEAD™ Viability/Cytotoxicity Kit - ROS and GSH level (DCFDA and CMFDA assay) - Intracellular activity of caspase 3/7 - Analysis of autophagy (Autophagy Sensors LC3B) - MMP-2 Assay	- 2 h - 24 h	CAD/CAM samples (HUGE dental PMMA blocks) displayed good biocompatibility during the 24 h exposure.	3D-printed materials were less biocompatible than CAD/CAM blocks. A significant decrease in the GSH level was detected in cells after incubation with MA-based 3D-printed samples, which indicated induction of oxidative stress.
Sultan 2024 [25]	1. **Katana Kuraray** (Noritake, Tokyo, Japan) 2. **IPSe.maxCAD** (Ivoclar Vivadent, Schaan, Liechtenstein) 3. **Grandio Disc** (VOCO, Cuxhaven, Germany)	No data	**Human gingival fibroblasts** (American Type Culture Collection ATCC, Manassas, VA, USA)	- MTT assay - Microscopic analysis	- 24 h - 48 h - 72 h	Zirconia (IPS e.maxCAD) emerges as a favorable option due to its minimal cytotoxic effects. Resin-based composites should be used cautiously, considering their higher cytotoxic potential.	Resin-based composites (Voco and Grandio Disc) showed the lower cell viability.

Glossary: MTT—(3-(4,5-Dimethyl-2-thiazolyl)-2,5-diphenyl-2H-tetrazolium bromide). MTS—(3-(4,5-dimethylthiazol-2-yl)-5-(3-carboxymethoxyphenyl)-2-(4-sulfophenyl)-2H-tetrazolium). ALP—Alkaline phosphatase. XTT—Yellow tetrazolium salt. CCK-8—Cell Counting Kit-8. LDH—Lactate Dehydrogenase. HGF—Human gingival fibroblasts. EDX—Energy dispersive X-ray. ELISA—Enzyme-linked immunosorbent assay. FACS—Fluorescence-activated cell sorter. CLSM—Confocal laser scanning microscopy. SEM—Scanning electron microscopy. UPLC–MS/MS—Ultra high-performance liquid chromatography coupled to mass tandem spectrometry. PGE2—Prostaglandin E2. IL—Interleukins. TNF-α—Tumor necrosis factor α.

**Table 3:** Summary findings for the primary outcome.

Outcome Significance	Author and Year	Quality of Evidence (GRADE)
No cytotoxicity of CAD/CAM materials	Tete 2014 [19]	+++− moderate due to imprecision
	Grenade 2016 [26]	++++ high
	Pabst 2016 [20]	++++ high
	Grenade 2017 [44]	++++ high
	Lee 2017 [52]	+++− moderate due to risk of bias
	Wang 2017 [45]	+−−− low due to imprecision and risk of bias
	Contreras 2018 [21]	++−− moderate due to imprecision
	Rizo−Gorrita 2018 [22]	+++− moderate due to indirectness
	Atay 2019 [36]	++−− low due to imprecision and indirectness
	Park 2019 [37]	+++− moderate due to imprecision
	Aydin 2020 [24]	++−− moderate due to imprecision and indirectness
	Campaner 2020 [38]	++++ high
	Cui 2020 [46]	++−− low due to imprecision and indirectness
	Souza 2020 [39]	++++ high
	Chen 2022 [47]	++++ high
	Mavriqi 2022 [28]	+++− moderate due to imprecision and indirecness
	Nakai 2022 [29]	++−− low due to imprecision and indirectness
	Wei 2022 [40]	++++ high
	Wuersching 2022 [50]	++++ high
	Aydin 2023 [41]	+++− moderate due to imprecision and indirecness
	Ille 2023 [30]	++−− low due to imprecision, risk of bias and indirectness
	Kim 2023 [31]	++−− low due to imprecision and risk of bias
	Ko 2023 [42]	+++− moderate due to indirectness and risk of bias
	Matsuura 2023 [32]	+++− moderate due to indirectness
	Rodríguez−Lozano 2023 [48]	+++− moderate due to indirectness
	Wei 2023 [43]	+++− moderate due to indirectness
	Kim 2024 [49]	+++− moderate due to imprecision
	Pantea 2021 [27]	+++− moderate due to indirectness and imprecision
	Saramet 2024 [34]	+++− moderate due to indirectness
Confirmed cytotoxicity of CAD/CAM materials	Hussain 2017 [35]	+++− moderate due to imprecision
	Shishehian 2018 [23]	++−− low due to imprecision, risk of bias and indirectness
	Ozverel 2023 [33]	+++− moderate due to indirectness
	Sultan 2024 [25]	++−− low due to imprecision and indirectness

## Data Availability

No new data were created or analyzed in this study. Data sharing is not applicable to this article.
